# Epigenetic response in mice mastitis: Role of histone H3 acetylation and microRNA(s) in the regulation of host inflammatory gene expression during *Staphylococcus aureus* infection

**DOI:** 10.1186/1868-7083-6-12

**Published:** 2014-06-30

**Authors:** Rahul Modak, Susweta Das Mitra, Madavan Vasudevan, Paramanandhan Krishnamoorthy, Manoj Kumar, Akshay V Bhat, Mani Bhuvana, Sankar K Ghosh, Bibek R Shome, Tapas K Kundu

**Affiliations:** 1 Transcription and Disease Laboratory, Molecular Biology and Genetics Unit Jawaharlal Nehru Centre for Advanced Scientific Research, Jakkur P.O, Bangalore 560064, India; 2 Project Directorate on Animal Disease Monitoring and Surveillance, Bangalore, India; 3 Bionivid Technology [P] Ltd, 401 - 4 AB Cross, 1st Main, Kasturi Nagar, East of NGEF, Bangalore 560043, India; 4 Department of Biotechnology, Assam University, Silchar 788011, India; 5 School of Biotechnology, Kalinga Institute of Industrial Technology, Campus XI, Patia, Bhubaneswar 751024, India; 6 Department of Biochemistry, Veterinary College and Research Institute, Namakkal 637002, India

**Keywords:** Epigenetic modifications, Histone, Acetylation, Mastitis, *S. aureus* infection, Micro-RNA, Gene expression, Inflammatory response, Chromatin immunoprecipitation

## Abstract

**Background:**

There is renewed interest towards understanding the host-pathogen interaction in the light of epigenetic modifications. Although epithelial tissue is the major site for host-pathogen interactions, there is handful of studies to show how epithelial cells respond to pathogens. Bacterial infection in the mammary gland parenchyma induces local and subsequently systemic inflammation that results in a complex disease called mastitis. Globally *Staphylococcus aureus* is the single largest mastitis pathogen and the infection can ultimately result in either subclinical or chronic and sometimes lifelong infection.

**Results:**

In the present report we have addressed the differential inflammatory response in mice mammary tissue during intramammary infection and the altered epigenetic context induced by two closely related strains of *S. aureus*, isolated from field samples. Immunohistochemical and immunoblotting analysis showed strain specific hyperacetylation at histone H3K9 and H3K14 residues. Global gene expression analysis in *S. aureus* infected mice mammary tissue revealed a selective set of upregulated genes that significantly correlated with the promoter specific, histone H3K14 acetylation. Furthermore, we have identified several differentially expressed known miRNAs and 3 novel miRNAs in *S. aureus* infected mice mammary tissue by small RNA sequencing. By employing these gene expression data, an attempt has been made to delineate the gene regulatory networks in the strain specific inflammatory response. Apparently, one of the isolates of *S. aureus* activated the NF-κB signaling leading to drastic inflammatory response and induction of immune surveillance, which could possibly lead to rapid clearance of the pathogen. The other strain repressed most of the inflammatory response, which might help in its sustenance in the host tissue.

**Conclusion:**

Taken together, our studies shed substantial lights to understand the mechanisms of strain specific differential inflammatory response to *S. aureus* infection during mastitis. In a broader perspective this study also paves the way to understand how certain bacteria can evade the immune surveillance and cause sustained infection while others are rapidly cleared from the host body.

## Background

Mastitis, characterized by inflammation of the mammary gland, is a major disease affecting dairy cattle worldwide [1]. The severity of the inflammation is dependent on the causative agent and the host response to it. Although viruses, fungi, or protozoa can cause mastitis, the most common causes are Gram positive and negative bacteria. These bacterial infections can result in a spectrum of clinical outcomes ranging from acute and life-threatening to chronic and subclinical mastitis. *Staphylococcus aureus* and *Escherichia coli* are the two largest single pathogens isolated from milk. *S. aureus* induced mastitis is characterized by a more moderate and delayed Somatic Cell Count (SCC) increase and infection can ultimately result in subclinical, chronic, and sometimes lifelong infection [2]. Outcome of infection can be influenced by the infecting strain [3]. *S. aureus* potentially produces a variety of virulence factors that contribute to persistence and pathogenicity of the organism [4]. Lipotechoic acid (LTA) is the major immune stimulatory molecule, identified in Gram positive organisms recognized by TLR-2. Activation of the pattern recognition receptors (PRR) initiates signaling cascades and finally leads to activation of NF-κB and MAP kinase pathways that culminate in the transcription of a wide range of immune genes including cytokines which are synthesized by infiltrating cells as well as resident cells in response to *S. aureus* infection [1,5]. We have selected two predominant evolutionarily related but not identical clones of *S. aureus* from fresh milk samples collected from subclinical cases of mastitis cows [6] to compare the host immune response during mastitis.

Bovine mammary epithelial cells (MEC) is a good model system to study host immune response during bacteria-induced mastitis, but recent reports have shown that it poorly mimics whole animal data [7]. Inbred mice strains, like BALB/c, have been extensively used to study mastitis. Our previous study had shown that *E. coli* infection induced severe mastitis in mice [8], which inspired us to use the same model system to study *S. aureus* induced mastitis. The alteration of global mRNA profile during *S. aureus* induced mastitis in bovine and goat has been reported earlier [9,10]. Recently Lawless *et al.*[11] reported alteration of microRNA (miRNA) profile in MECs upon *Streptococcus uberis* infection. However the alteration of miRNA upon *S. aureus* infection in the host mammary tissue has not been studied. Vanselow *et al.*[12] reported DNA remethylation at the alphaS1-casein promoter during *E. coli* infection induced bovine mastitis. Wang *et al.*[13] also showed aberrant DNA methylation at the CD4 gene promoter in the peripheral blood cells of the mastitis cow.

In the present study we have developed a mice model for *S. aureus* induced mastitis. *S. aureus* infection induced hyperacetylation at histone H3K9 and H3K14 residues in mice mammary tissue. Hyperacetylated histones were selectively enriched at the promoters of overexpressed proinflammatory genes. *S. aureus* infection also altered the microRNA profile of the host tissue. We have used two *S. aureus* isolates to compare the strain specific response of mice mammary tissue. Remarkably, it was observed that the two strains of *S. aureus* induced differential expression of inflammatory genes. The strain relative/specific alteration in gene expression in mice tissue seems to be linked to the histone acetylation pattern. The RNA sequence analysis of the infected tissue revealed that small microRNAs expression also changed upon infection, suggesting a complex network of coding and non-coding RNA expression (in the context specific epigenetic state) in the process of pathogen clearance or sustained infection.

## Results

### *Staphylococcus aureus* is the major mastitis pathogen isolated from milk samples

**Figure 1:**
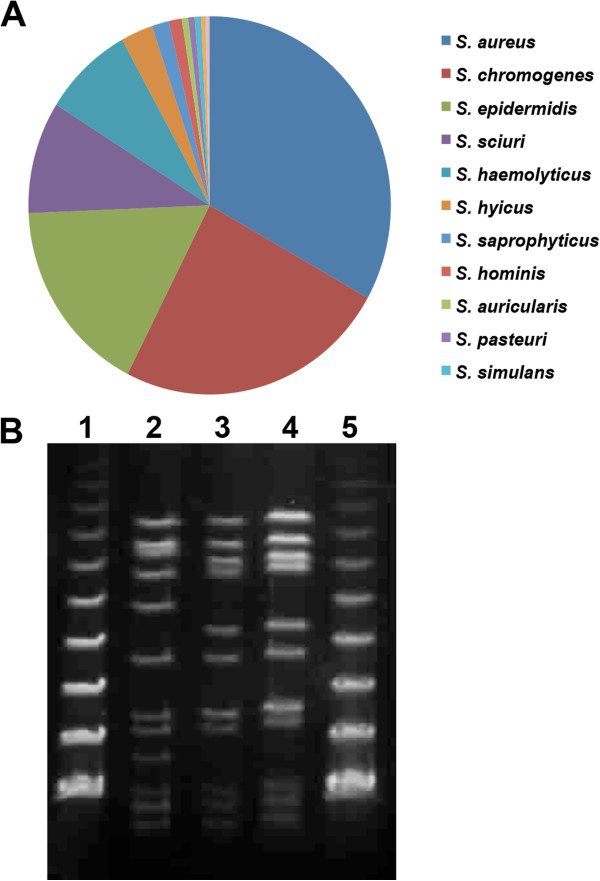
**Isolation and characterization of different *staphylococcus *species. (A)** Distribution of *Staphylococcus* species inbovine milk sample. **(B)** Pulsed field gel electrophoresis (PFGE) banding pattern of SmaI digests of genomic DNA of *Staphylococcus aureus*. Lanes 1 and 5, PFGE lambda DNA marker; Lane 2, *S. aureus* strain SA1 (t6877); Lanes 3 and 4, *S. aureus* strain SA2 (t267).

In order to identify the different bacterial pathogens present in the milk, we collected 294 fresh milk samples from subclinical cases of mastitis cows reared in seven independent farms. 705 different bacterial isolates were classified into different species by 16S rRNA sequencing and species specific PCR [14] and *Staphylococcus sp.* emerged as the major mastitis pathogen isolated from these samples [6]. The majority of the isolates could be grouped under the subclass of coagulase negative staphylococci (CONS), which are the largest emerging class of mastitis pathogens all over the world. In these samples we found that *S. aureus* is the largest single pathogenic species (Figure 1A, Table 1). Genetic diversity among these *S. aureus* isolates has been reported earlier [6]. We had checked the presence of eight major virulence factors and most of the isolates were found to be positive for at least five different virulence genes. In order to establish *S. aureus* induced mastitis in mice, we selected two isolates harboring seven virulence genes. These two isolates, HF-14LY/t6877 and HF-16Y/t-267 (designated as SA1 and SA2, respectively), belong to different pulsotypes (Figure 1B) as revealed by pulse-field gel electrophoresis (PFGE).

**Table 1:** **Distribution of identified *Staphylococcus *** **spp. from bovine milk samples**

***Staphylococcus*** **species**	**Total species identified**		**Total no. submitted to gene bank**	**Gene bank accession no.**
*S. aureus*	173	33.14%	101	HM452003-2094
*S. chromogenes*	127	24.33%	89	HM367743-7826; HM452095-2099
*S. epidermidis*	88	16.86%	57	HM367827-7875; HM452104-2111
*S. sciuri*	50	9.58%	43	HM451958-1991; HM451994-1999; HM452101-2103
*S. haemolyticus*	42	8.05%	34	HM359218-9243;HM452114-2121
*S. hyicus*	15	2.87%	13	HM451942-1954
*S. saprophyticus*	8	1.53%	8	HM451933-1938; HM452112-2113
*S. hominis*	6	1.15%	5	HM451928-1932
*S. auricularis*	3	0.57%	3	HM451939-1941
*S. pasteuri*	3	0.57%	3	HM451955-1956
*S. simulans*	3	0.57%	3	HM452000; HM452100; HM462053
*S. gallinarium*	2	0.38%	2	HM452122-2123
*S. warneri*	1	0.19%	1	HM452001
*S. devreisi*	1	0.19%	1	HM452124

### Mice mammary tissue differentially responds to *S. aureus* infection

**Figure 2:**
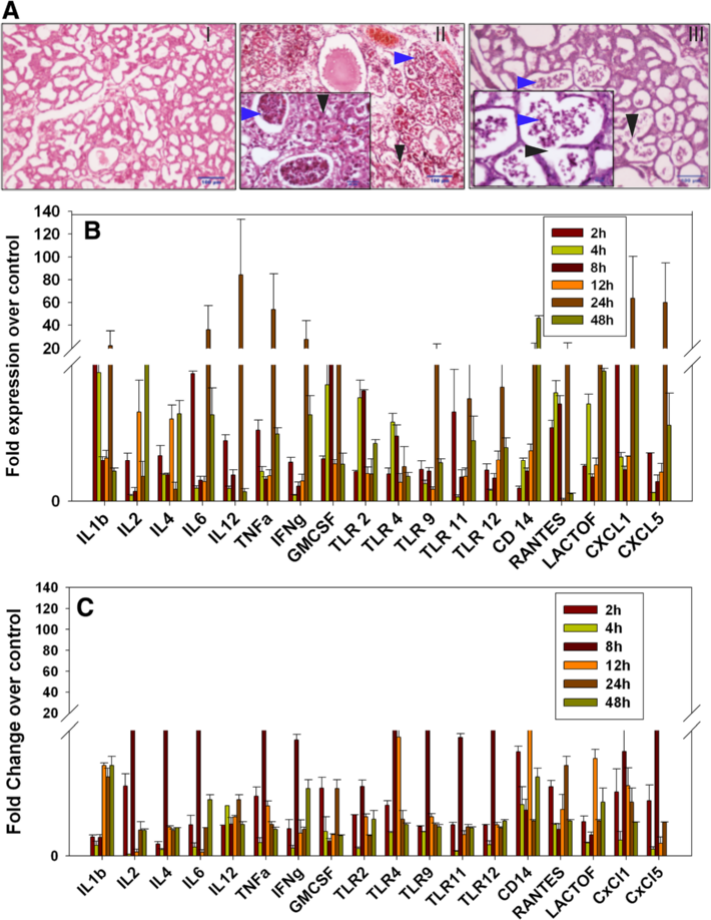
***S. aureus *infection induces mastitis in mice mammary tissue. (A)** Comparative histopathological analysis of PBS (I) *versus S. aureus* (II and III) inoculated mice mammary tissue showed clear induction of mastitis after 24 h of infection. SA1 (panel II) inoculated tissue showed more pronounced infiltration of macrophages and neutrophils (blue arrow) in the alveolar lumen, than SA2 (panel III). Black arrow indicates tissue necrosis. **(B,C)** Temporal expression of various genes in infected tissue against PBS control was analyzed by qRT-PCR. Fold overexpression of the genes in *S. aureus* infected tissue over the control tissue was calculated and plotted as average of three biological replicates. SA1 **(B)** infection consistently showed higher inflammatory response compared to SA2 **(C)** at all time points.

Gross examination of *S. aureus* infected mice with both SA1 and SA strains showed redness, congestion, and swollen mammary glands at 24 and 48 hpostinoculation. The PBS inoculated gland showed the mammary gland engorged with milk and appeared normal. On histopathological examination, the PBS inoculated mice showed alveoli filled with milk proteins and lipid droplets and the presence of few alveolar macrophages in interalveolar space and perialveolar space were seen in both the control mammary glands. Histopathological analysis of formalin fixed tissues revealed the establishment of *S. aureus* induced mastitis in a temporal manner in case of both SA1 and SA2 infection. The SA1 inoculated mice on histological examination showed a few alveolar macrophages and neutrophils in the alveolar lumen after 8 h of inoculation. At 12 h, the alveolar epithelial cells showed thinning and dilatation of alveoli with moderate infiltration of polymorpho nuclear and mononuclear cells in alveolar lumen and interalveolar space. Upon progression, the alveolar epithelial cells showed vacuolar degeneration (Figure 2A-II, black arrow) and neutrophils and mononuclear cells occupying the entire lumen was observed 24 h post inoculation (Figure 2A-II, blue arrow). At 48 h, there was loss of alveolar architecture, severe infiltration of mononuclear cells with fat cells and severe damage to the mammary gland tissues were observed (Additional file 1: Figure S1A and Additional file 2: Figure S1B).

The SA2 inoculated mice showed no observable histological changes after 2, 4, and 8 h of intramammary inoculation. At 12 h, mild infiltration of alveolar macrophages in the interstitial space with vacuolar degeneration of alveolar epithelial cells was observed. After 24 h the alveolar lumen showed moderate infiltration of neutrophils and mononuclear cells (Figure 2A-III, blue arrow) with necrosis of alveolar epithelial cells were observed. The adjacent alveoli coalesce to form large alveoli and thinning of alveolar epithelial cells was also observed (Figure 2A-III, black arrow). After 48 h of inoculation, severe infiltration of mononuclear cells, occupying the entire alveolar lumen and interalveolar space along with necrosis of the alveolar epithelial cells was observed. The loss of alveolar architecture was seen with few areas showing intact alveoli and few fat cells were observed. Based on the histopathological changes observed in the mammary glands, the SA1 caused more severe histological changes when compared to SA2 infected mice (Additional file 1: Figure S1A and Additional file 2: Figure S1B).

Gram-positive bacterium like *S. aureus* stimulates innate immune response through a wide variety of bacterial components like peptidoglycan, lipoteichoic acid, lipopeptides, and bacterial DNA. These pathogen associated molecular patterns (PAMPs) bind to TLR2 and other PAMP receptors and activate MyD88 complex mediated signalling cascades like classical NF-κB, MAPK, phosphoinositide 3-kinase (PI3K)/AKT pathways [15]. Activation of TLR2-MyD88 cascade leads secretion of several pro-inflammatory mediators like interleukins, interferon (IFN)γ, TNFα, and cytokines that are essential for the recruitment and survival of polymorphonuclear neutrophils (PMN). Quantitative RT-PCR (qRT-PCR) analysis of a set of pro-inflammatory genes had shown gradual increase in their expression from 4 h to 24 h post infection by both SA1 and SA2 (Figure 2B,C). Interestingly, at 48 h post infection most of these genes showed significant decrease in expression level that is essential for sustained *S. aureus* infection. We also observed markedly higher expression of pro-inflammatory genes in SA1 infected samples in comparison to SA2 infected tissues. This further highlights the differential host tissue response to different pathogen strains.

### *S. aureus* infection induces specific histone H3 hyperacetylation in alveolar epithelial cells

**Figure 3:**
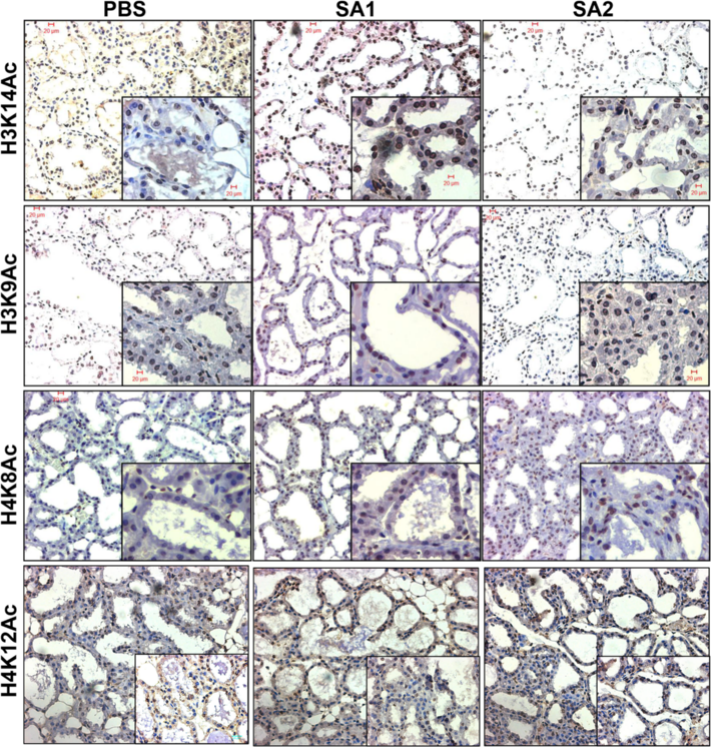
***S*. *aureus *infection induces histone hyperacetylation in mice mammary tissue.** Representative images of immunohistochemical analysis of mice mammary tissue (20× magnification, inset 40× magnification) stained with antibodies are indicated on the left side of the panel.

**Figure 4:**
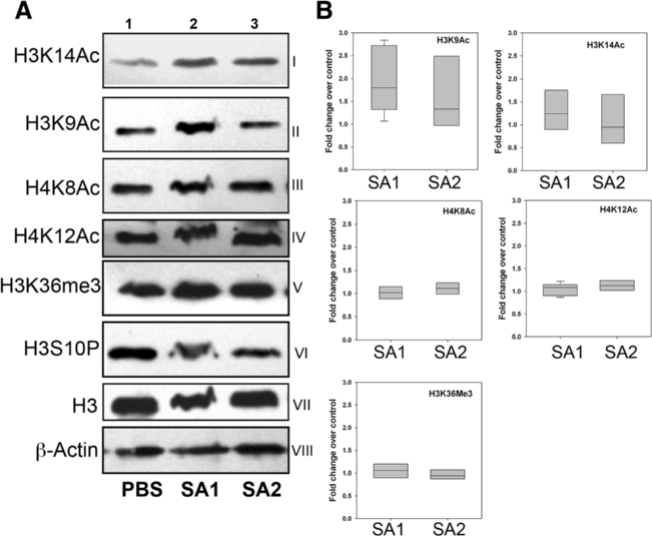
***S. aureus *infection specifically induces histone H3K9 and H3K14-acetylation in mice. (A)** SA1 and SA2 inoculated mouse mammary tissues were analyzed by western blots using antibodies specific to acetylated H3K9 (I), H3K14 (II), H4K8 (III), and H4K12 (IV), methylated H3K36 (V), and phosphorylated H3S10 (VI). The levels of histone H3 (VII) and β-actin (VIII) were used as loading controls. Lane 1, PBS inoculated, Lane 2, SA1 inoculated and Lane 3, SA2 inoculated mouse mammary tissue. **(B)** Densitometric analysis of western blots. Western blots were scanned by Bio-Rad Geldoc XR scanner and band intensities were quantified by Quantity One™ software. Each lane was independently compared with PBS control after normalization with H3. The box-plots show median band intensities of three independent western blots.

Bacterial infection induced histone hyperacetylation in the inflammatory cells is well documented. Our earlier study for the first time has shown that *E. coli* infection induces histone hyperacetylation in the mammary epithelial cells [8]. Immunohistochemical (IHC) analysis of the *S. aureus* infected mice mammary tissue showed hyperacetylation at histone H3K9 and H3K14 residues in the mammary epithelial cells (Figure 3, Additional file 3: Figure S2A and Additional file 4: Figure S2B). Significantly we observed that apart from H3K9 and H3K14 other acetylation marks (H4K8, H4K12) were minimally altered upon SA1 and SA2 infection. However, between SA1 and SA2, substantial difference could be noticed even at the context of H3K9 and H3K14 acetylation. In order to further confirm the alteration of different histone acetylation marks upon SA1 and SA2 infection in comparison to PBS control, we extracted the total histones from control and infected tissue and subjected to western blot analysis. The result closely resembles the IHC data such as significant hyperacetylation at H3K9 and H3K14 residues could be noticed in the SA1 infected tissue (compare Lane 1 *vs.* Lanes 2 and 3 of panels 1 and 2, Figure 4A). However, no significant difference could be found for H3S10 phosphorylation, H3K36 trimethylation as well as H4K8 and H4K12 acetylation (compare Lane 1 *vs.* Lanes 2 and 3 panels 4 to 6, Figure 4A). The western blotting data from multiple independent experiments were also quantified using densitometric scanning (Figure 4B, Additional file 5: Figure S3). An approximate two-fold increase in H3K9 acetylation was observed upon SA1 infection in mice mammary tissues whereas very modest change in the level of acetylation could be observed due to SA2. SA1 infected samples also showed a 1.3-fold increase in H3K14ac (Figure 4B), whereas its level in SA2 infected samples was not statistically significant. Collectively, these data clearly suggest that the acetylation of H3 at K9 and K14 substantially altered upon *S. aureus* (Strain SA1 and SA2) infection and more predominantly the acetylation of H3K14 was induced.

### *S. aureus* infection induces differential expression of coding and non-coding (miRNA) transcripts

**Figure 5:**
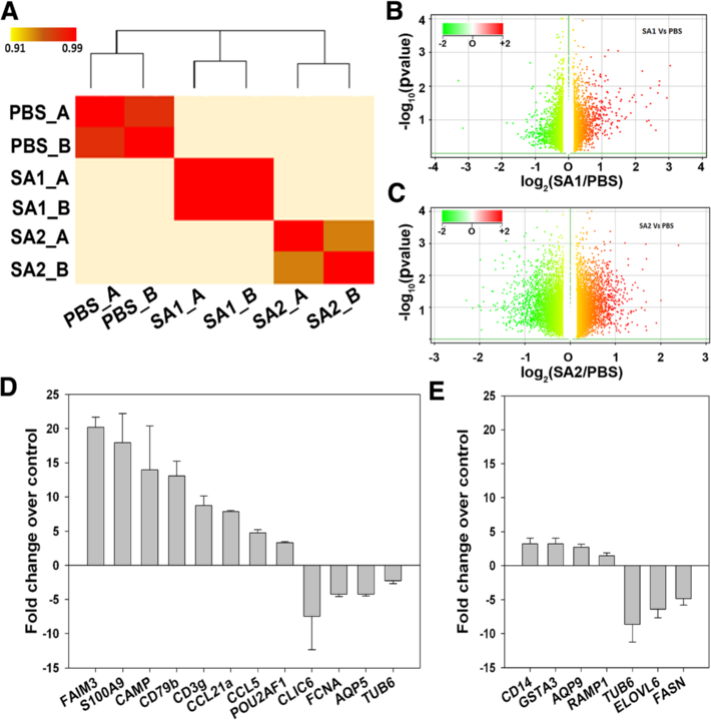
***Staphylococcus aureus *infection induces strain specific alteration of global gene expression in mice mammary tissue. (A)** Expression heat map of sample-to-sample distances on the matrix of variance-stabilized data for overall gene expression. Darker red colors indicate more similar expression (color key is in arbitrary units). Clustering (top) demonstrates that the biological replicates of PBS, SA1 and SA2 samples are similar to each other but show complete separation among individual groups. **(B)** Differential gene expression, with fold difference between log2 normalized expression in SA1 inoculated (*n* = 2) and PBS inoculated mice mammary tissue (*n* = 2) plotted *versus*-log10 adjusted *P*value. Each gene is colored on the basis of the log10 base mean expression. **(C)** Differential gene expression, with fold difference between log2 normalized expression in SA2 inoculated (*n* = 2) and PBS inoculated mice mammary tissue (*n* = 2) plotted *versus*-log10 adjusted *P *value. Each gene is colored on the basis of the log10 base mean expression. **(D,E)** Expression of a set of genes in the SA1 **(D)** or SA2 **(E)** infected mice mammary tissue was analyzed by qRT-PCR. The fold difference in expression of the genes in infected tissue over the PBS control tissue was calculated and plotted as the average of three biological replicates for SA1 and two biological replicates for SA2.

In order to elucidate the global alteration of gene expression profile upon the infection and its possible link with epigenetic modifications we isolated the total RNA from the infected tissue and first subjected to microarray analysis using Illumina Mouse WG6 BeadChip (v3) array (GSE54232). Two sets of biological replicate samples, each comprising PBS treated, SA1 and SA2 infected were subjected to microarray analysis (Figure 5). We then independently compared differential expression of genes between SA1 and SA2 infected samples with the PBS control group using a negative binomial test [16]. In SA1 infected tissue, 43 genes were differentially expressed 1.5-fold or more and *P* < 0.05 after false discovery rate (FDR) correction (Figure 5B). In SA2 infected tissue 151 genes were differentially expressed 1.5-fold or more and *P* < 0.05 after FDR correction (Figure 5C). Interestingly, we observed SA1 infection induced significant overexpression of a small number of pro-inflammatory genes, interleukins and cytokines, whereas SA2 infection mostly downregulated expression of many genes to a moderate extent. These data indicate that two closely related strains of *S. aureus* could elicit very different inflammatory responses in mice mammary tissue. These observations were further validated by quantitative real-time PCR (qRT-PCR) (Figure 5D,E) that clearly shows SA1 infection induced drastic (five-fold and above) overexpression of a set of proinflammatory genes, whereas SA2 infection moderately (less than five-fold) altered the gene expression.

As RNA Polymerase-II-driven transcription also synthesize small non-coding RNAs, including microRNAs (miR), we further selected the small RNAs from the total RNA isolated from the PBS, SA1 and SA2 (one each) inoculated mice mammary tissue. cDNA library was prepared for these samples and then sequenced through next generation sequencing (NGS). After performing the initial quality check the small RNAseq data were analyzed through a workflow (Additional file 6: Figure S4) that included mapping sequences to different small RNA annotation, identifying differentially expressed microRNAs and determining the mRNA targets.

**Figure 6:**
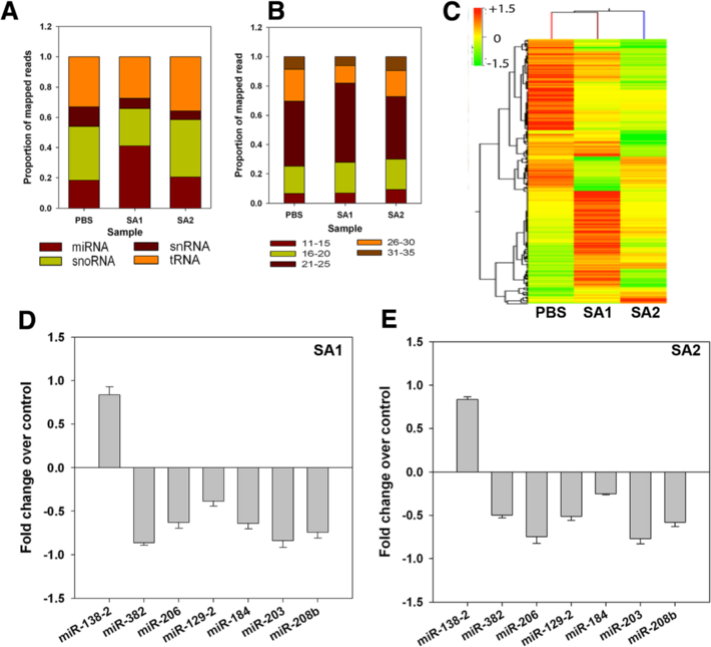
***S*.*aureus *infection alters global small RNA expression profile in mice mammary tissue in a strain specific manner. (A)** Global distribution of small RNA transcripts. Short reads were sequentially mapped to sequences of transfer RNAs (tRNA), small nuclear RNAs (snRNA), small nucleolar RNAs (snoRNAs), and microRNA (miRNA) precursor sequences of mouse reference genome. **(B)** Length distribution of miRNA short reads in all three samples. Reads were adaptor trimmed. Reads <10 nt were discarded and reads >40 nt were shortened to 40 nt by clipping from the 3’ end. **(C)** Expression heat-map showing the alteration in global miRNA expression in the mice mammary tissue after 24 h of *S. aureus* infection. Clustering (top) demonstrates that the PBS, SA1, and SA2 samples show complete separation. **(D,E)** Expression of a set of miRNAs in the SA1 **(D)** or SA2 **(E)** Infected mice mammary tissue was analyzed by qRT-PCR. The fold difference in expression of the genes in infected tissue over the PBS control tissue was calculated and plotted as the average of three biological replicates for SA1 and two biological replicates for SA2.

We obtained 22.6 million adaptor-trimmed reads of average read length of 11 to 35 nt from three mouse mammary samples. After removal of rRNA reads, the reads were further classified into microRNA (miR), tRNA, small nuclear RNA (snRNA), and small nucleolar RNA (snoRNA) (Figure 6A). All the three samples had comparable numbers of tRNA reads. In comparison to PBS and SA2 infected samples, SA1 showed presence of a very high number of miRs and a low number of snoRNAs. The miRNAs were further classified based on tag length and a large abundance of 21 to 25 nt long reads was observed (Figure 6B). The expression of microRNAs showed broad dynamic range. We used DESeq for further statistical analysis of the data [17]. We observed 41 and 18 miRNAs are differentially expressed (above 1.5-fold) in SA1 and SA2 infected tissues respectively (Figure 6C). Interestingly SA1 infection induced both up- and downregulation of miRNAs, whereas SA2 infection induced repression of 18 miRNAs. The complete lists of DE microRNAs and their expression values have been included in Additional file 7: Table S1. The DE miRNAs were validated by qRT-PCR (Figure 6D,E). The validated data confirm that the selected set of microRNAs is indeed differentially expressed in the infected tissue.

In order to understand the functions of these DE miRNAs we performed correlation analysis with mRNA expression data. We used three different algorithms, Targetscan, miRANDA, and PITA [18-20], to predict microRNAs complementary to 3’-untranslated regions (3’UTRs) of the DE mRNA in our data. Then we compared these predicted miRNAs to our DE microRNAs and identified annotated and novel DE miRNAs. We have identified 6 and 23 of anti-correlated DE miRNAs and mRNAs in SA1 and SA2 infected tissues, respectively, through negative correlation analysis. Interestingly many of the DE mRNA and miRNA did not show any correlation, which indicates involvement of other regulatory mechanisms of gene expression. As expected there was large variation in the number of predicted interactions of microRNAs and mRNAs (Additional file 8: Table S2).

**Figure 7:**
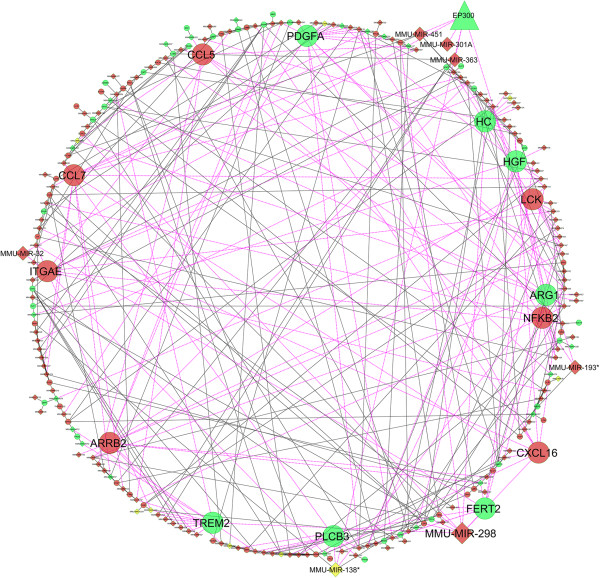
***S. aureus *infection alters the expression of several genes and miRNAs in the mice mammary tissue.** Biological network analysis showing the differential expression of various genes involved in immune response and their regulatory miRNAs in the SA1 infected mice mammary tissue. Genes that are overexpressed compared to PBS are shown in red, and repressed genes are marked in green. Genes known to play a critical role in inflammatory and immune response are shown in a bigger size. The lines connecting overexpressed nodes are shown in red and downregulated nodes are shown in green.

**Figure 8:**
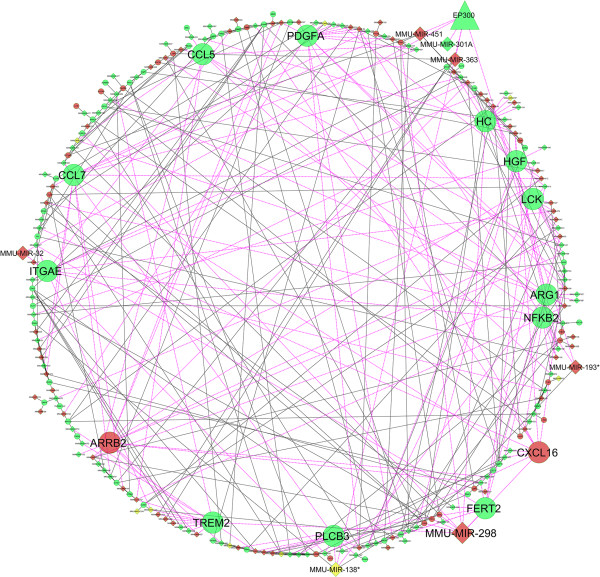
***S. aureus *infection alters the expression of several genes and miRNAs in the mice mammary tissue.** Biological network analysis showing the differential expression of various genes involved in immune response and their regulatory miRNAs in the SA2 infected mice mammary tissue. Genes that are overexpressed compared to PBS are shown in red, and repressed genes are marked in green. Genes known to play a critical role in inflammatory and immune response are shown in a bigger size. The lines connecting overexpressed nodes are shown in red and downregulated nodes are shown in green.

In-depth analysis of mRNA-miRNA interactome reveals significant interactions and pathways (Figures 7 and 8). Both SA1 and SA2 infected tissue showed reduced expression of protein lysine acetyltransferase (KAT) p300 (KAT3B) mRNA but no change in the expression of other known KATs (GCN5/PCAF, Tip60, and so on) could be observed. We could not check p300 protein levels in these samples due to technical limitations. We speculate that there should be enrichment of acetylated p300 that in turn induced H3 acetylation. H3K9 hyperacetylation in the *S. aureus* infected mice tissue also indicates plausible role of PCAF (KAT2B), which could be explored further. Reduction of p300 mRNA was concomitant with increased expression of mmu-miR-451 and mmu-miR-363 that targets 3’-UTR of p300. Mmu-miR-451 is known to destabilize cytokine mRNAs [21] and mmu-miR-363 is known to be associated with regulation of MAPK signaling [22]. SA1 infected tissue had high levels of mmu-miR-301 that represses NF-κB repressing factor (NRF) [23] and led to induction of NF-κB (Figure 7). In SA2 infected tissue there was reduction in both mmu-miR-301 and NF-κB (Figure 8). Both SA1 and SA2 infection led to expression of mmu-miR-298 that targets IKKi/IKKϵ [24] and thus regulates NF-κB pathway [25]. Upregulation of mmu-miR-150 (Additional file 7: Table S1) upon SA1 infection will negatively regulate CIITA and thereby help in immune evasion [26]. Overexpression of mmu-Let7 miRNA will directly inhibit IL6 [27] and which could be the cause of rapid decrease in IL6 expression. Upregulated mmu-miR-20 family could be useful in chromatin remodeling and subsequent gene expression. mmu-miR-20 and mmu-miR-106 have to regulate IL10 expression and macrophage inflammatory response [28,29]. All these data clearly suggest that SA1 infection induces overexpression of a bunch of microRNAs that is known to target mRNA of genes pervasively involved in proinflammatory responses. SA2 infection induced expression of genes like Trem2 (triggering receptor expressed on myeloid cells), FERT2 (Fer tyrosine kinase-2) that will suppress inflammatory response (Figure 8) [26,30]. SA2 infection negatively regulated expression of genes like b-arrestin (ARRB2 [31]), integrinAϵ (ITGAE [32]), hemolytic complement (HC [33]), lymphocyte protein tyrosine kinase (LCK [34]) that are involved in pathogen clearance or induction of immune response. To our surprise SA1 infection had completely opposite effects on the expression of these genes that makes it susceptible to immune surveillance and subsequent clearance from the host. Collectively these data suggest that strain specific differential gene expression due to *S. aureus* infection*,* is regulated by epigenetic response.

### Histone H3K14 acetylation is selectively enriched at the overexpressed gene promoters

**Figure 9:**
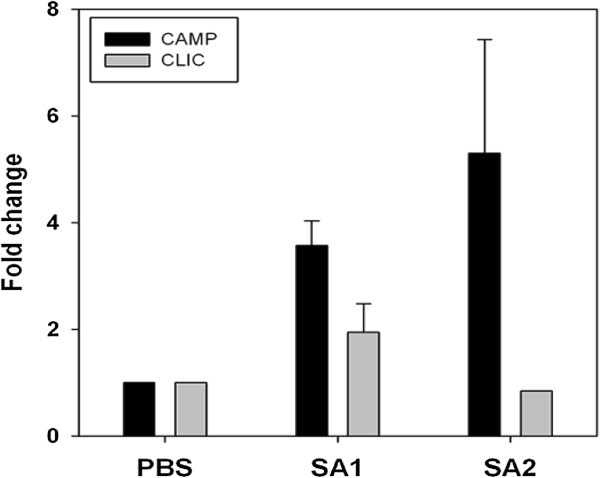
***S. aureus *induces selective enrichment of H3K14 acetylation at specific gene promoters.** Acetylated H3K14 are selectively enriched at the promoters of the immune genes upon SA1 infection in the mice mammary tissue. ChIP assays were done with acetylated H3K9, H3K14, and H4K8 antibodies along with IgG controls in the tissue sections of *S. aureus* infected mice mammary tissue. The fold difference in expression of the genes in infected tissue over the PBS control tissue was calculated and plotted as the average of three biological replicates for SA1 and two biological replicates for SA2.

Gram-positive bacterial cell wall components have been shown to activate TLR-2 receptors on the host cells that in turn activate NF-κB signaling. We had performed chromatin-immunoprecipitation (ChIP) assay with the formalin fixed paraffin embedded (FFPE) tissues collected at 24 h post infection. We observed selective enrichment of H3K14 acetylation at the CAMP gene promoter in both SA1 and SA2 infected tissue but not at CLIC gene promoter (Figure 9). Microarray data suggested that CAMP was significantly upregulated in SA1 infected tissue whereas there was downregulation of CLIC (Figure 5B,D). We have not seen any enrichment of histone H3K9 and H4K8 acetylation in any of the promoter. These data clearly show that there is selective enrichment of acetylated histone H3 at upregulated gene promoter.

## Discussion

Mastitis is a complex disease-involving multitude of host and pathogen factors. The outcome of bacterial infection ranges from unnoticeable subclinical mastitis to life-threating severe infection. The growth and pathogenesis of any bacterium is largely influenced by the host immune system but the detailed understanding of pathogenesis in mastitis is sketchy. Our previous studies have shown that staphylococci are the largest pathogen group associated with mastitis [14], which is as per with the global scenario [35]. We have observed that *S. aureus* is the largest single mastitis pathogen isolated from fresh cow milk samples (Figure 1A and Mitra *et al.*[6]). *S. aureus* infection induces less virulent subclinical mastitis that persists for a long time. Recently it has been shown that *E. coli* and *S. aureus* induced mastitis elicit very different inflammatory responses, both in mammary tissue and in MECs [7,36,37].

In the present study we have successfully established *S. aureus* induced mastitis in mice. Our data clearly demonstrate that two closely related strains of *S. aureus* induced very different inflammatory responses in mice mammary tissue (Figure 2A). At 24 h post infection, SA1 infected tissue showed drastic induction in inflammatory genes expression (Figure 2B), which is associated with infiltration of neutrophils and alveolar macrophages. SA2 infection induced significantly lower inflammatory response in the mice mammary tissue (Figure 2C) and there were fewer numbers of macrophages. Interestingly, in both cases the expression of inflammatory genes drastically decreased at 48 h post infection that is contrary to our previous observations in the case of *E. coli* induced mastitis [8]. This is not surprising, given the fact that *S. aureus* infection often leads to asymptomatic but sustained mastitis whereas *E. coli* infection often gets cleared rapidly. This observation encouraged us to investigate the regulatory mechanisms of gene expression during *S. aureus* induced mastitis.

Forty-three genes were differentially expressed in the SA1 (fold change ≥2, FDR ≤0.05) (Figure 5B) infected tissue and 151 genes were dysregulated in SA2 infected tissue (Figure 5C). Similar numbers of genes were differentially expressed during *S. aureus* induced mastitis in goat and cow [9,36], but their expression levels were different. The number of differentially expressed genes in *S. aureus* induced mastitis in mice are significantly lower than *E. coli* infection [8], which had been reported in bovine mastitis as well [10]. Detailed analysis of gene expression data showed differential response between SA1 and SA2 infection (Figure 2B,C, Figure 5). Downregulation of tubulin expression indicated induction of tissue damage that was supported by histopathological analysis (Figure 2A). All these data clearly indicated that *S. aureus* infection induced various signaling pathways in mice mastitis. We have decided to study the role of epigenetic mechanisms in the regulation of gene expression during *S. aureus* induced mastitis in mice.

Alteration of miRNA expression during bacterial infection has been well documented. Lawless *et al.*[11] reported differential expression of a set of microRNAs in bovine MEC upon *Streptococcus uberis* infection. Alteration of small RNA in *S. aureus* and its role in gene expression has been extensively studied [38,39]. There are few reports to show the alteration of host microRNA profile by *S. aureus* cell wall components [2,40]. Recently Jensen *et al.*[36] had shown that *S. aureus* infection alters the gene expression profile of the uninfected quarter in bovine mastitis which underscores the necessity for the whole animal- live pathogen model to study host-pathogen interactions. To our knowledge there is no study to show the effect of live *S. aureus* infection on the host small RNA profile.

SA1 infection led to a two-fold increase in the total number of expressed microRNAs (Figure 6A), whereas SA2 did not significantly alter the number of microRNAs. There was no notable difference in the size distribution of miRNAs in all three samples indicating absence of size bias upon treatment (Figure 6B). DE analysis showed SA1 infection both induced as well as repressed expression of several microRNAs, whereas SA2 infection mostly led to repression of microRNA expression (Figure 6C). For detailed analysis of gene expression and miRNA expression data please refer to Additional file 9.

Immuno-histochemical analysis (Figure 3) coupled with western blotting (Figure 4) showed significant hyperacetylation at histone H3K9 and H3K14 residues in the SA1 infected tissue, whereas there was hardly any difference between SA2 and PBS inoculated tissues. Unlike *E. coli* infection [8], there was no change in histone H4 acetylation and H3K36 trimethylation levels during the *S. aureus* induced mastitis. We did not observe any change in the H3S10 phosphorylation in the *S. aureus* infected tissue, which is contrary to previous reports during other Gram-positive bacterial infection [41]. Taken together these data indicate that there are few epigenetic modifications (like H3K14 acetylation) that are commonly altered during many bacterial infections. It also strongly argues for the fact that there is pathogen specific alteration of other epigenetic modifications, which clearly highlights the complexity of the epigenetic regulation associated with the tissue response during bacterial infection. Selective enrichment of histone H3K14 acetylation at the overexpressed gene promoter (Figure 9) clearly shows involvement of histone modifications in the process. We have not detected significant enrichment of H3K14ac in many other promoters that could be due to overall low levels of histone hyperacetylation in the infected tissue. Although western blot and IHC analysis showed H3K9 hyperacetylation in SA2 infected tissue, we did not see any promoter specific enrichment of H3K9 acetylation. These data clearly shows that *S. aureus* infection induced both global as well as promoter specific H3K14 hyperacetylation, whereas H3K9 acetylation levels had been altered globally but may not be in a promoter specific manner. It is also possible that poor efficacy of the H3K9ac antibody in formaldehyde-fixed paraffin embedded (FFPE) ChIP applications interfered in detection of promoter specific enrichment. Our data argue for the fact that hyperacetylation of H3K14 residue is necessary but not sufficient for global transcription activation. A combination of several histone modifications (Figure 3 and Modak *et al.*[8]) writes the epigenetic language of global transcription activation during bacterial infection.

In summary, we have demonstrated that *S. aureus* infection induces specific histone posttranslational modifications during mice mastitis. It also induces differential expression of a set of microRNAs during mice mastitis. Our data clearly show the strain specific alteration of epigenetic modulators, namely histone acetylation and microRNA expression, that results in differential expression of pro- and anti-inflammatory genes. This kind of strain specific alterations in host gene expression will subject some pathogens for rapid clearance whereas others can sustain for longer duration inside the host. This probably could partially explain why *S. aureus* vaccine has not been successful so far. Although the mechanism of differential inflammatory response is not very evident from this study, yet we speculate the involvement of activated p300 and/or PCAF/GCN5 in the entire process. This study also shows the role of host microRNAs in host-pathogen interaction and disease manifestation. In future these epigenetic modifications can emerge as potential therapeutic target for mastitis and/or other bacterial diseases.

## Conclusion

Mastitis is one of the major bacterial infection induced pathogenic condition that affects all mammals including human. Globally bovine mastitis is the biggest challenge to the dairy industry that causes a financial burden of several million dollars. *E. coli* and *S. aureus* are the two major mastitis pathogens. In the present study we show that *S. aureus* infection altered host gene expression in a strain dependent as well as temporal manner. The change in host gene expression is regulated via alteration in a specific set of histone posttranslational modifications and microRNA expression. All these data indicate that *S. aureus* infection creates an environment favorable for sustenance of pathogen in the host tissue in a strain specific manner. Collectively these data show how bacterial infection alters host epigenetic landscape and provide new insights into epigenetic regulation of bacterial strain specific host inflammatory response.

## Methods

### Virulence gene profiling of *S. aureus* isolates

The genus and species-specific PCR conditions has been reported earlier [14]. All the *S. aureus* isolates were screened for the presence of eight major virulence factors-*fnb*A, *fnb*B, *clf*A, *cna*, *pvl*, *tst*, *nuc*, and *coa*, in their genome. The detailed PCR amplification condition has been reported earlier [6].

### PFGE of *S. aureus* isolates

Two *S. aureus* isolates,SA1-HF-14Y/t6877 and SA2-HF-16LY/t-267-each harboring seven virulent genes, were selected for further characterization by pulse field gel electrophoresis (PFGE) following the protocol published by Mitra *et al.*[6].

### Ethics statement

The animal experiments have been approved by Institutional Animal Ethics Committee (IAEC) of PD_ADMAS where the animal experiments were carried out bearing Committee for the Purpose of Control and Supervision of Experiments on Animals (CPCSEA) registration no. 881/03/ac/05/CPCSEA. The animal experiments protocol was approved during the IAEC meeting held on 22 October 2011 by the committee members. The animal experiments were carried out as per the guidelines of CPCSEA, Government of India, New Delhi.

### Establishment of mastitis in Swiss albino mice

Timed pregnant Swiss albino mice procured from National Centre for Laboratory Animal Science (NCLAS), National Institute of Nutrition, Hyderabad, India were acclimatized under controlled conditions in individually ventilated cages (IVC).

Inoculum dose: The most predominant clones of *S. aureus* field isolates (SA1 and SA2) were selected based on characterization profile to challenge the mice. The*S. aureus* was grown in brain heart infusion (BHI) broth overnight and centrifuged at 3,000 × g for 5 min at room temperature to pellet the cells. *S. aureus* pellet was washed with sterile cell culture tested PBS (HiMedia) twice to remove all the media components and finally resuspended in PBS. Standard plate count (SPC) method was employed to adjust the bacterial load at a cell density of 5 × 10^3^ cfu/ 50 μL for intramammary inoculation.

Inoculation procedure:*S. aureus* challenge experiments were performed following the protocol described by Chandler [42-44] with modifications. Briefly, 7 days postpartum mice were anesthetized with intraperitoneal injection of 2 mg Ketamine per mouse. Mice teats were cleansed with 70% ethanol. A total of 50 μL of the bacterial culture were carefully inoculated with a sterile syringe fitted with 30G needle in two abdominal pairs of mammary glands (left fourth (L4), right fourth (R4), left fifth (L5), and right fifth (R5)) in 36 mice. Twelve mice were simultaneously inoculated with PBS as control. Mice recovered from anesthesia were left in the cage with pups to suckle 1 h post inoculation. The animals were examined at regular intervals for generalized and local reaction that include redness of the teat, swelling of mammary gland, and appearance of a sunken abdomen.

Tissue collection: Mice were sacrificed with overdose of Ketamine and laid on their back; the abdominal skin was opened by mid ventral incision. The mammary glands were carefully dissected out using sterile scissor and forceps at 2, 4, 8, 12, 24, and 48 h post inoculation. Gross examination of mammary gland tissues was carried out and was collected in protease inhibitor cocktail (Sigma), RNA- later® solution (Ambion), and 10% formalin (Merck). The samples collected in RNA-later were incubated at 4°C overnight. RNA stabilized tissues were flash frozen in liquid nitrogen after discarding the RNA-later and stored at -80°C. The formalin fixed tissues were processed for histopathology, stained with hematoxylin and eosin and were observed under light microscope for recording histological changes. The same tissues were used for immunohistochemical analysis following the protocol published by Modak *et al.*[8]. Tissues collected in protease inhibitor were incubated on ice for 1 h and then flash frozen before storing at -80°C till further processing. For western blot analysis, tissue lysates were prepared following the previously published protocol [8].

### RNA isolation, microarray, and small RNA sequencing

Total RNA was isolated from mouse mammary tissues using TRIzol® Reagent (Invitrogen) as per manufacturer’s instructions. RNA preparations were stored at -80°C till further use. The RNA samples were further processed for genes expression analysis by microarray and small RNA analysis by deep sequencing. For detailed procedure please refer to the Additional file 9.

### Computational analysis of sequencing and microarray data

#### Small RNA sequencing

Small 35 nt RNA reads were produced using an IlluminaGAIIx Genome Analyzer. The detailed methodologies for the microarray and small RNA data processing and pathway analysis have been included in Additional file 9.

#### Integration of microarray and small RNA sequencing data

miRNA: mRNA integrome was performed by using differentially expressed miRNA and its target genes. Further, miRScape plugin (http://ferrolab.dmi.unict.it/mirscape.html) for Cytoscape v 8.0 was used to visualize the enriched miRNA: mRNA integrome to understand the key regulatory circuits.

### Real time qRT-PCR analysis

qRT-PCR to study the temporal expression of inflammatory genes was performed using gene specific probes (Roche) (Additional file 7: Table S1) using Roche Lightcycler® 480 real-time PCR system. Total mRNA present in the samples was converted to cDNA by First Strand cDNA Synthesis Kit (Thermo Scientific) using oligo-dT primer. Primer3 software was used to design gene specific primers for qRT-PCR (Additional file 8: Table S2). Total RNA isolated from mice mammary tissue were converted to cDNA by NCode™ VILO™ miRNA cDNA Synthesis Kit (Invitrogen). For qRT-PCR, miRNA specific forward primers (Additional file 10: Table S3) were designed as per manufacturer’s protocol and universal qPCR supplied with the kit was used as reverse primer. SensiFAST SYBR No-ROX Kit (Bioline) was used for qRT-PCR.

### Chromatin immunoprecipitation (ChIP) assay

ChIP assays were performed with the FFPE mice mammary tissue samples using H3K9ac, H3K14ac, and H4K8ac antibodies. The detailed ChIP- qRT-PCR protocol has been reported earlier [8]. Primers to amplify the promoters of the selective genes have been listed in Additional file 9: Table S4. NCBI GEO accession no.: GSE54230, GSE54231, GSE54232.

## Abbreviations

ChIP: Chromatin Immunoprecipitation; FFPE: Formalin Fixed Paraffin Embedded; IHC: immunohistochemistry/Immunohistochemical; miRNA: microRNA; qRT PCR: Quantitative Real Time Polymerase chain reaction; SDS-PAGE: Sodium dodecyl sulphate polyacrylamide gel electrophoresis.

## Competing interests

The authors declare no competing financial interests.

## Authors’ contributions

TKK, BRS, and RM were responsible for overall conceptualization of the study and writing manuscript. RM and SDM initiated the study and performed majority of the experiments. SDM, MB, and PK were involved in collection and isolation and characterization of pathogens and mice experiments. BRS and SKG guided pathogen characterization and mice experiments. MK was associated with ChIP assays. AB was associated with IHC analysis. RM and MV analyzed all the microarray and small RNA sequencing data. All authors read and approved the final manuscript.

## Supplementary Material

Additional file 1: Figure S1A Induction of *S. aureus* induced mastitis in mice mammary tissue. Comparison of SA1 vs. PBS inoculated mice mammary tissue histopathological sections from 2 h to 48 h post infection. Scale bar shows 100 μm. Click here for file

Additional file 2: Figure S1B Induction of *S. aureus* induced mastitis in mice mammary tissue. Comparison of SA2 vs. PBS inoculated mice mammary tissue histopathological sections from 2 h to 48 h post infection. Scale bar shows 100 μm. Click here for file

Additional file 3: Figure S2A Alteration of histone acetylation in the mice mammary tissue upon *S. aureus* infection. Representative images of immunohistochemical analysis of mice mammary tissue (20× magnification, inset 40× magnification). Antibodies are indicated on the top of the panel. Biological replicates for each set of treatment (PBS, SA1 and SA2) have been arranged in columns, which are indicated below. Scale bar 20 μm. Click here for file

Additional file 4: Figure S2B Alteration of histone acetylation in the mice mammary tissue upon *S. aureus* infection. Representative images of immunohistochemical analysis of mice mammary tissue (20× magnification, inset 40× magnification). Antibodies are indicated on the top of the panel. Biological replicates for each set of treatment (PBS, SA1 and SA2) have been arranged in columns, which are indicated below. Scale bar 20 μm. Click here for file

Additional file 5: Figure S3 *Staphylococcus aureus* infection specifically induces histone H3K9 and H3K14 -acetylation in mice. SA1 and SA2 inoculated mouse mammary tissues were analyzed by Western blots using antibodies specific to acetylated H3K9, H3K14, H4K8 and H4K12, methylated H3K36 and phosphorylated H3S10. The levels of histone H3 and β-actin were used as loading controls. Lane 1, 4, 7, PBS inoculated (biological replicates), Lane 2, 5, 8, SA1 inoculated (biological replicates) and Lane 3, 6, SA2 inoculated (biological replicates) mouse mammary tissue. Click here for file

Additional file 6: Figure S4 Schematic representation of small RNA data analysis workflow including integration of mRNA microarray data. Click here for file

Additional file 7: Table S1 SA1 and SA2 inoculated mouse mammary tissues were analyzed by Western blots using antibodies specific to IL6 (I) and IFNγ (II) and Tubulin (III) were used as loading controls. Lane 1, PBS inoculated, Lane 2, SA1 inoculated and Lane 3, SA2 inoculated mouse mammary tissue. Click here for file

Additional file 8: Table S2 Details of differentially expressed microRNAs in the *S. aureus* infected mice mammary tissue. Click here for file

Additional file 9 Supplementary Results & Discussion and Methods. Click here for file

Additional file 10: Table S3 List of differentially expressed miRNAs and expression levels of their known targets in the *S. aureus* infected mice mammary tissue. Click here for file
