# Supplementary figures and images for: Epigenetic response in mice mastitis: Role of histone H3 acetylation and microRNA(s) in the regulation of host inflammatory gene expression during Staphylococcus aureus infection
Source: Clin Epigenetics. 2014 Jun 30;6(1):12. doi: 10.1186/1868-7083-6-12 (PMC4114167; doi:10.1186/1868-7083-6-12)

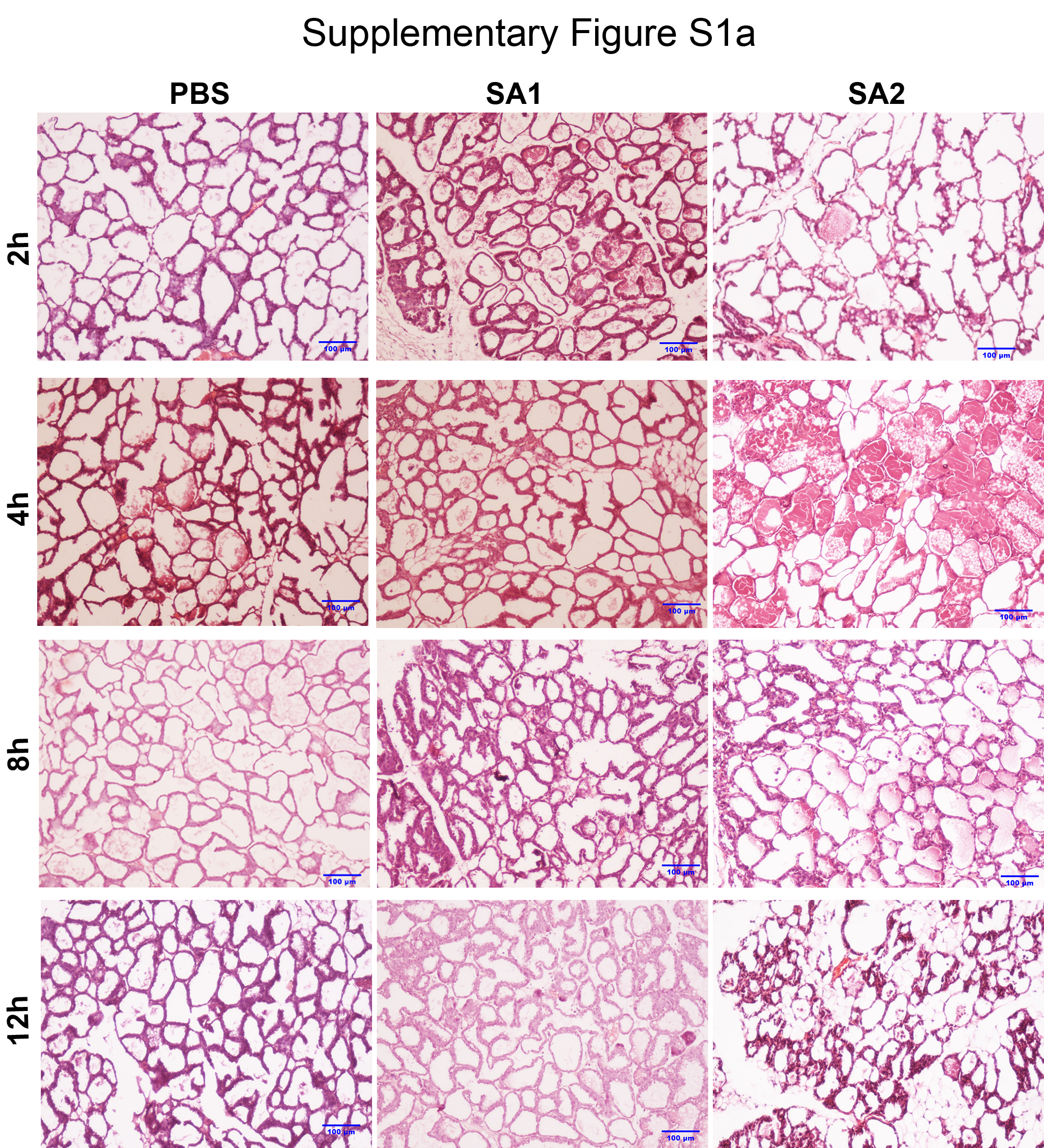

Supplement: Additional file 1: Figure S1A — Induction of S. aureus induced mastitis in mice mammary tissue. Comparison of SA1 vs. PBS inoculated mice mammary tissue histopathological sections from 2 h to 48 h post infection. Scale bar shows 100 μm. [file 1868-7083-6-12-S1.tiff]

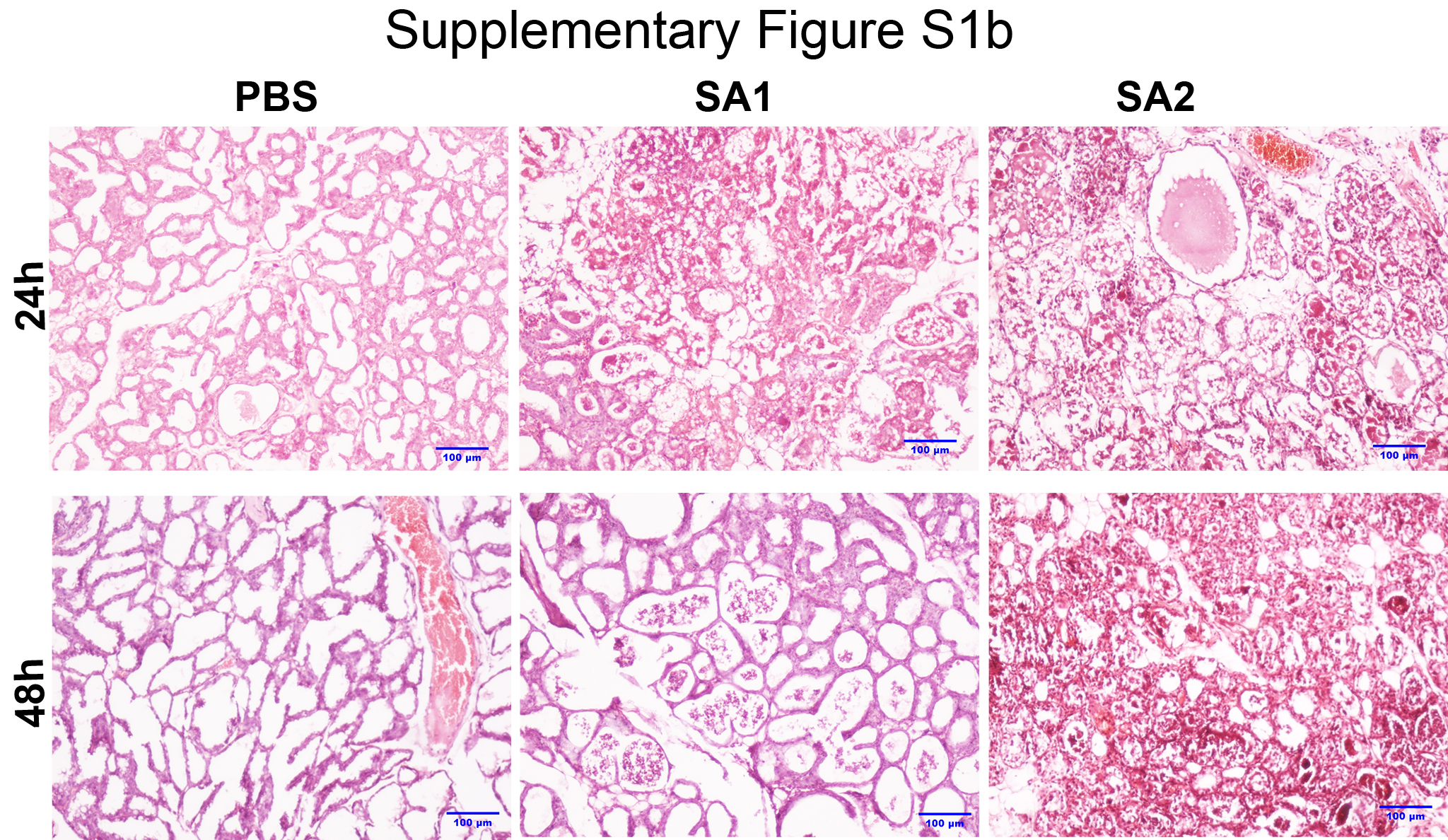

Supplement: Additional file 2: Figure S1B — Induction of S. aureus induced mastitis in mice mammary tissue. Comparison of SA2 vs. PBS inoculated mice mammary tissue histopathological sections from 2 h to 48 h post infection. Scale bar shows 100 μm. [file 1868-7083-6-12-S2.tiff]

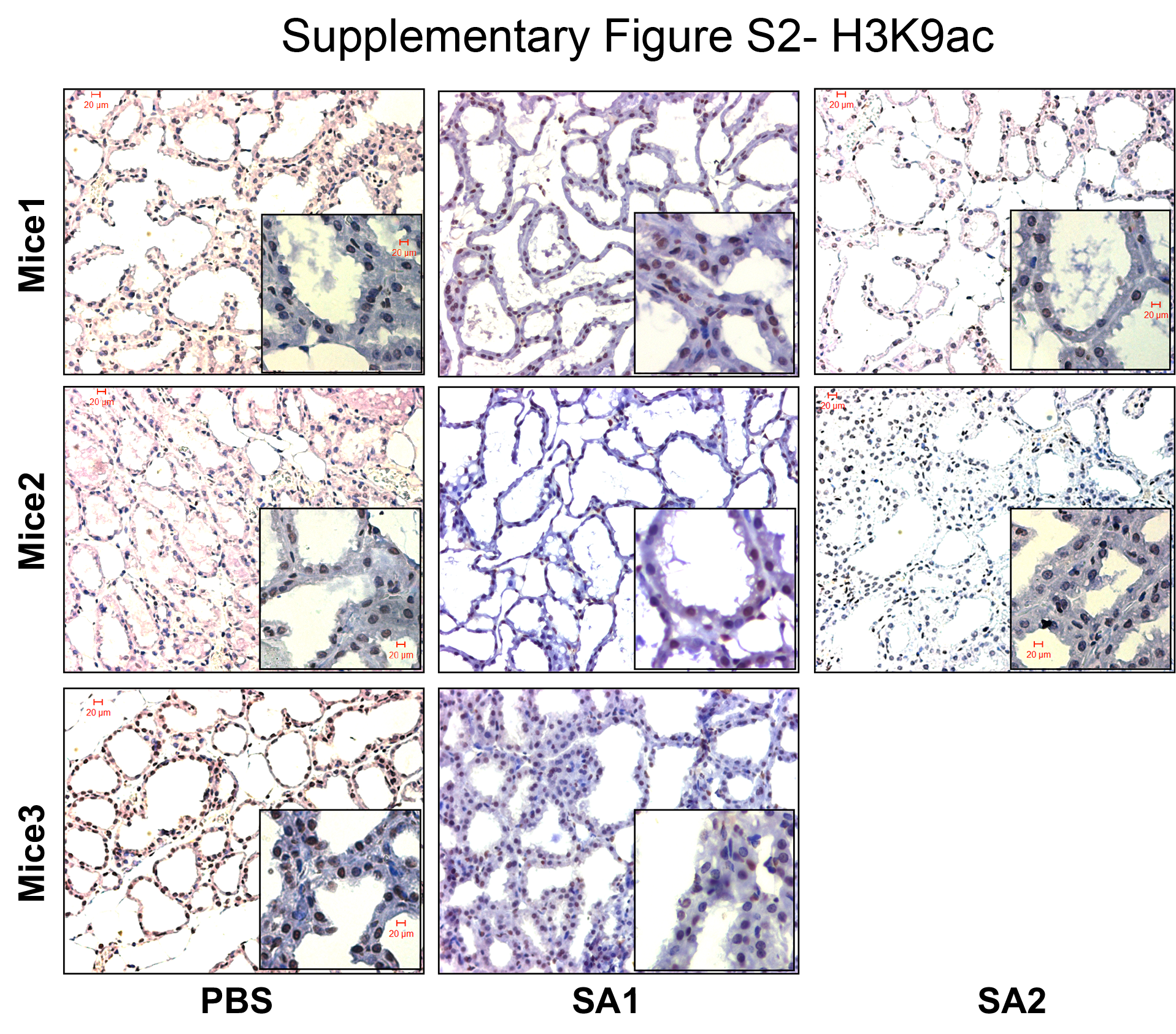

Supplement: Additional file 3: Figure S2A — Alteration of histone acetylation in the mice mammary tissue upon S. aureus infection. Representative images of immunohistochemical analysis of mice mammary tissue (20× magnification, inset 40× magnification). Antibodies are indicated on the top of the panel. Biological replicates for each set of treatment (PBS, SA1 and SA2) have been arranged in columns, which are indicated below. Scale bar 20 μm. [file 1868-7083-6-12-S3.tiff]

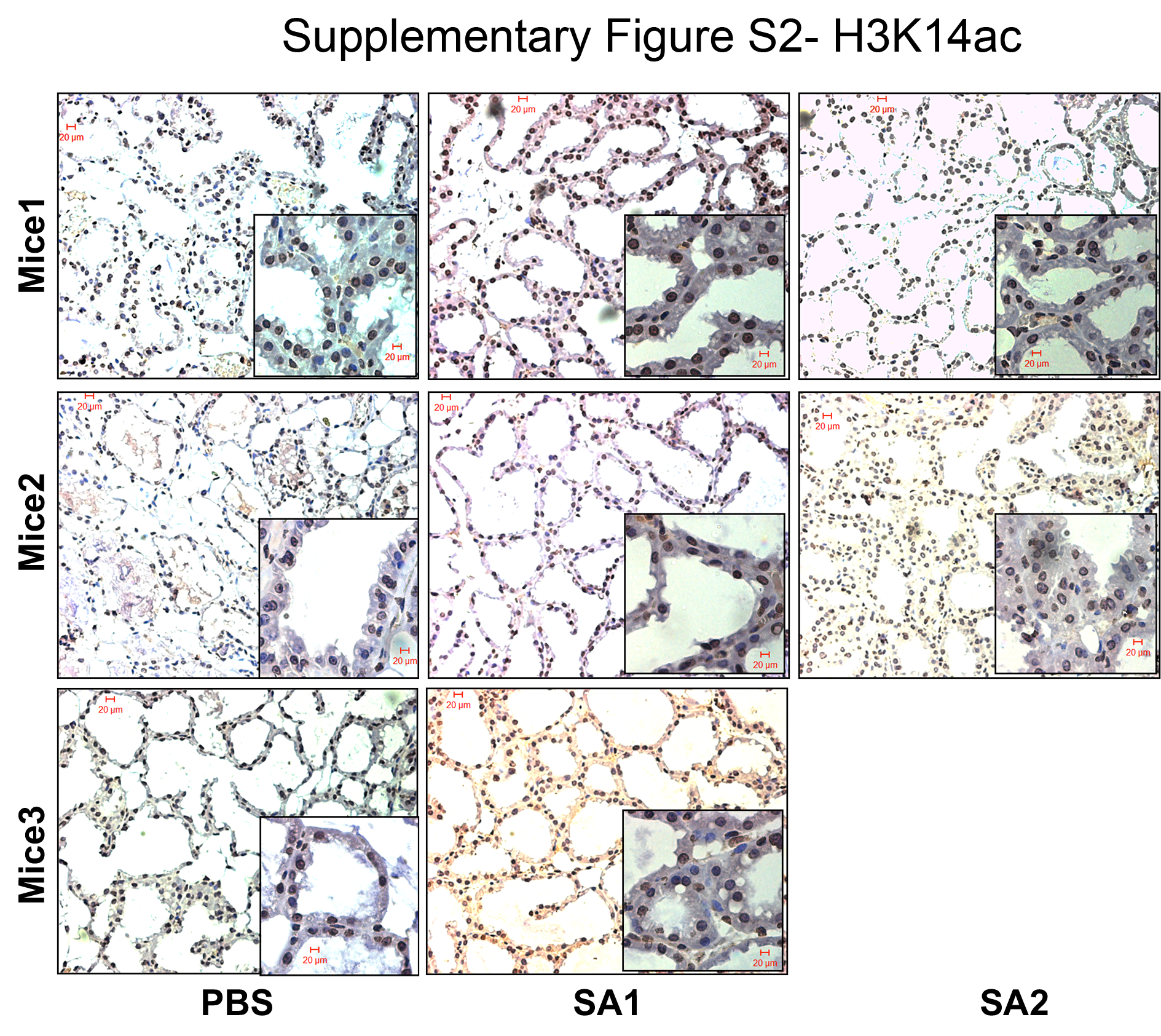

Supplement: Additional file 4: Figure S2B — Alteration of histone acetylation in the mice mammary tissue upon S. aureus infection. Representative images of immunohistochemical analysis of mice mammary tissue (20× magnification, inset 40× magnification). Antibodies are indicated on the top of the panel. Biological replicates for each set of treatment (PBS, SA1 and SA2) have been arranged in columns, which are indicated below. Scale bar 20 μm. [file 1868-7083-6-12-S4.tiff]

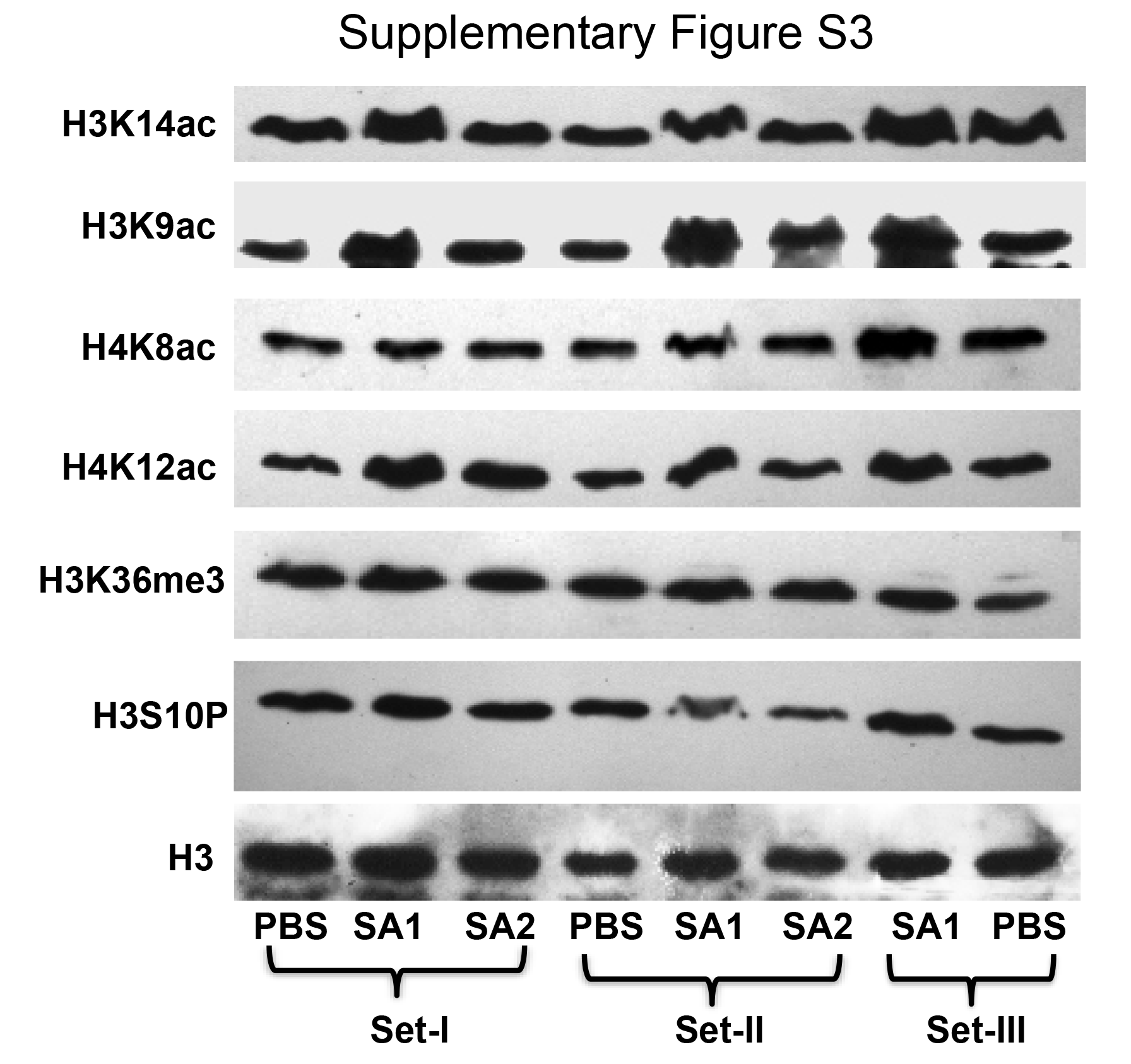

Supplement: Additional file 5: Figure S3 — Staphylococcus aureus infection specifically induces histone H3K9 and H3K14 -acetylation in mice. SA1 and SA2 inoculated mouse mammary tissues were analyzed by Western blots using antibodies specific to acetylated H3K9, H3K14, H4K8 and H4K12, methylated H3K36 and phosphorylated H3S10. The levels of histone H3 and β-actin were used as loading controls. Lane 1, 4, 7, PBS inoculated (biological replicates), Lane 2, 5, 8, SA1 inoculated (biological replicates) and Lane 3, 6, SA2 inoculated (biological replicates) mouse mammary tissue. [file 1868-7083-6-12-S5.tiff]

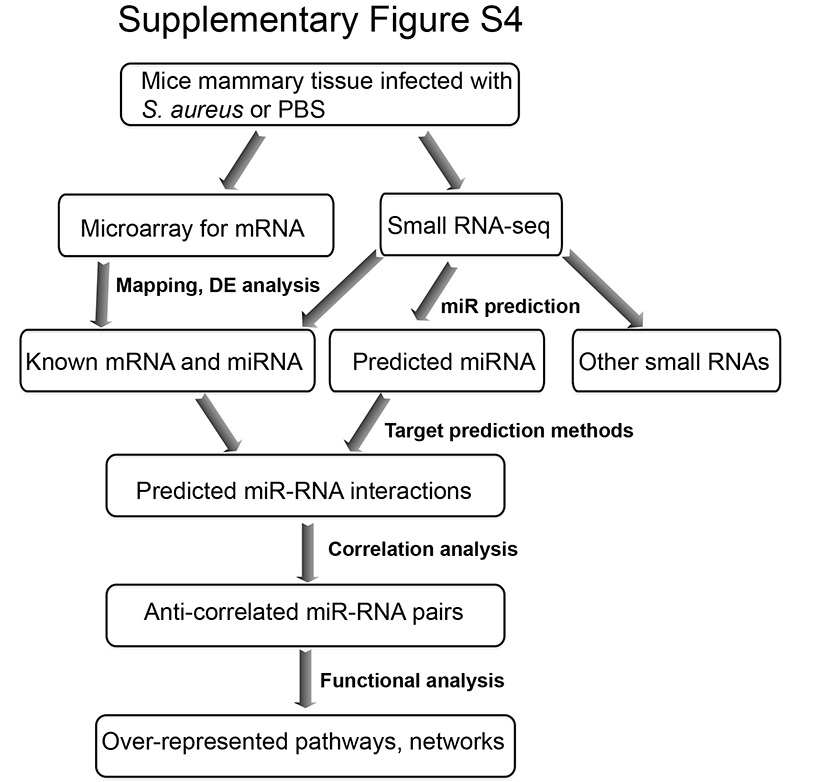

Supplement: Additional file 6: Figure S4 — Schematic representation of small RNA data analysis workflow including integration of mRNA microarray data. [file 1868-7083-6-12-S6.tiff]
