# Supplementary material for: Epigenetic response in mice mastitis: Role of histone H3 acetylation and microRNA(s) in the regulation of host inflammatory gene expression during Staphylococcus aureus infection
Source: Clin Epigenetics. 2014 Jun 30;6(1):12. doi: 10.1186/1868-7083-6-12 (PMC4114167; doi:10.1186/1868-7083-6-12)

**Supplemental Material to:**

Rahul Modak, Susweta Das Mitra, MadavanVasudevan, P. Krishnamoorthy, Manoj Kumar, AkshayBhat, M. Bhuvana, SankarK Ghosh, Bibek R. Shome and Tapas K. Kundu^*^

**Epigenetic response in mice mastitis: Role of Histone H3 Acetylation and microRNA(s) in the regulation of host inflammatory gene expression during *Staphylococcus aureus i*nfection**

**Supplementary Results**

43 genes were differentially expressed in the SA1 (fold change ≥2, FDR ≤ 0.05) (Figure 5B) infected tissue and 151 genes were dysregulated in SA2 infected tissue (Figure 5C). Similar numbers of genes were differentially expressed during *S. aureus* induced mastitis in goat and cow (9, 34), but their expression levels were different. The number of differentially expressed genes in *S. aureus* induced mastitis in mice are significantly lower than *E. coli* infection(8), which had been reported in bovine mastitis as well (10). Detailed analysis of gene expression data showed differential response between SA1 and SA2 infection (Figure 2 B-C, Figure 5). SA1 infection induced expression of several proinflammatory genes like interleukins (*IL1β, IL2,IL4, IL6, IL12* etc.), cytokines (*CCL5/RANTES, CXCL1, CXCL5, GMCSF*) and cell surface receptors like *CD14, TLR2, TLR4, TLR9* and *TLR12*. SA2 infections induced moderate over expression of all the proinflammatory genes and in both the cases the expression of these genes reduced significantly after 48h. There was no expression of anti-inflammatory genes like *IL10*, which indicates *S. aureus* infection regulates the inflammatory response through alternative mechanisms. SA1 infection induced expression of cathelicidin antimicrobial peptide (*Camp*) that plays key role in innate immune defense during bacterial infection (37-39). Both SA1 and SA2 downregulated expression of aquaporins that would help them to sustain in the mammary tissue. Downregulation of tubulin expression indicated induction of tissue damage that was supported by histopathological analysis (Figure 2A). All these data clearly indicated that *S. aureus* infection induced various signalling pathways in mice mastitis. We have decided to study the role of epigenetic mechanisms in the regulation of gene expression during *S. aureus* induced mastitis in mice.

Alteration of miRNA expression during bacterial infection has been well documented. Lawless et. al. (11) reported differential expression of a set of microRNAs in bovine MEC upon *Streptococcus uberis* infection. Alteration of small RNA in *S. aureus* and its role in gene expression has been extensively studied (40, 41). There are few reports to show the alteration of host microRNA profile by *S. aureus* cell wall components (2, 42). Recently Jensen et. al. (34) had shown that *S. aureus* infection alters the gene expression profile of the uninfected quarter in bovine mastitis which underscores the necessity for the whole animal- live pathogen model to study host-pathogen interactions. To our knowledge there is no study to show the effect of live *S. aureus* infection on the host small RNA profile.

SA1 infection led to 2-fold increase in the total number of expressed microRNAs (Figure 6A), whereas SA2 did not significantly alter the number of microRNAs. There was no notable difference in the size distribution of miRNAs in all the 3 samples indicating absence of size bias upon treatment (Figure 6B). DE analysis showed SA1 infection both induced as well as repressed expression of several microRNAs, whereas SA2 infection mostly led to repression of microRNA expression (Figure 6C). SA1 infection led to upregulation of microRNAs (e.g. mmu-miR-150, mmu-miR-106a, mmu-miR-146, mmu-miR-125, mmu-miR-155, mmu-Let7, mmu-miR-21 etc.), which could negatively regulate inflammatory response. We observed overexpression of 3 novel microRNAs- mmu-miR-1193, mmu-miR-669d and mmu-miR-1306, whose biological roles have not been studied yet. Most of these miRNAs are downregulated in SA2 infected tissue, indicating strain specific differential response of mice mammary tissues towards bacterial infection.

***S.aureus* infection induces IL6 and IFNγ expression in mice.**

Bacterial infection induced expression of inflammatory genes in the inflammatory cells is well documented. Our RT-PCR data showed overexpression of selective inflammatory response genes in the *S. aureus* infected mice mammary tissue (Figure 2 B-C). Western blot analysis of the infected tissue showed significant overexpression of IL6 and IFNγ in the SA1 infected tissue(Supplementary Figure S5). There was no change in the expression of IFNγ   infected tissue. These data reconfirms that *S. aureus* infection induces strain specific inflammatory response.

**Supplementary Methods**

**RNA isolation, microarray and small RNA sequencing**

Total RNA was isolated from mouse mammary tissues using TRIzol® Reagent (Invitrogen) as per manufacturer's instruction. RNA preparations were stored at - 80°C till further use.

Deep sequencing:For deep sequencing the small RNA samples were prepared as follows: total RNA of each sample was size-fractionated on a 15% PAGE gel, and a 16-30 nt fraction was collected. The 5' RNA adapter (5'-GUUCAGAGUUCUACAGUCCGACGAUC-3') was ligated to the RNA pool with T4 RNA ligase. Ligated RNA was size-fractionated on a 15% agarose gel, and a 40-60 nt fraction excised. The 3'RNA adapter (5'-pUCGUAUGCCGUCUUCUGCUUGidT-3'; p, phosphate; idT, inverted deoxythymidine) was subsequently ligated to precipitated RNA using T4 RNA ligase. Ligated RNA was size-fractionated on a 10% agarose gel, and the 70-90 nt fraction (small RNA + adaptors) excised. Small RNAs ligated with adaptors were subjected to RT-PCR (Superscript II reverse transcriptase, 15 cycles of amplification) to produce sequencing libraries. PCR products were purified and small RNA libraries were sequenced using IlluminaGAIIx, a massively parallel sequencing technology.

Microarray Processing: Biotin-labeled cRNA samples for hybridization were prepared according to Illumina's recommended sample labeling procedure: 500 ng of total RNA was used for cDNAsynthesis, followed by an amplification/labeling step (in vitro transcription) to synthesize biotin-labeled cRNA using the Illumina® TotalPrep RNA Amplification kit (Ambion Inc., Austin, TX). cRNA concentrations were determined by Nanodrop, ND-1000spectrophotometer (Thermo). Labeled, amplified material (750 ng per array) was hybridized to a ver. 3 of the Illumina Mouse WG6 BeadChip according to the Manufacturer's instructions (Illumina, Inc., San Diego, CA). Arrays were scanned with an Illumina Bead array Reader confocal scanner (BeadStation 500GXDW; Illumina, Inc., San Diego, CA) according to the Manufacturer's instructions.

**Computational analysis of sequencing and microarray data**

Small RNA sequencing: Small 35 nt RNA reads were produced using an IlluminaGAIIx Genome Analyzer. Low quality reads were trimmed using NGSQC Tool kit. After elimination of redundancy, sequences ≥ 18 nt were mapped to the mouse genomebuildMm9. Sequences that perfectly matched the genome along their entire length were considered for subsequent analyses. Genome sequences and annotations of the mouse genome (Mm9) were downloaded from NCBI. Sequences matching mouse rRNA, tRNA, snRNA and snoRNA deposited at the NCBI GenBank database or overlapping with rRNA and tRNA annotations of the genome were discarded. Repeat overlapping sequences were annotated as repeat-associated small RNAs. The majority of sequences overlapping with predicted exons were excluded from further analysis in view of the possibility that they were derived from messenger RNAs, and only those with precursors presenting p-values below 0.01 were collected.

After loading small RNAs and mapping information, small RNAs were sorted according to their position on the reference genome. Each read start position was examined to establish its similarity to a Drosha/Dicer processing site. For each candidate site, the mature sequence was extended to obtain two possible pre-miRNAs. One sequence encompassed 10 nt upstream and 70 nt downstream, and the other included 70 upstream and 10 downstream of the respective miRNAs. The two possible pre-miRNAs were evaluated using Mfold to determine ability to form characteristic hairpin structures. A well-designed computational filter was employed to identify miRNA-like hairpins. RNAs displaying folding energy values ≤ -25 kcal/mol were subjected to further analysis. Both miRNA and miRNA* had to reside in different arms of a hairpin structure, each with no more than 6 unpaired bases. We ensured that the maximum bulge over the miRNA/miRNA* duplex was not more than 4 bases, and asymmetry of the miRNA/miRNA* duplex equal to or less than 3. In addition to the above requirements, sequencing of both miRNA and miRNA* required that the miRNA/miRNA* duplex had 3' overhangs at both ends, a typical feature of Drosha and Dicer processing. Candidates corresponding to known miRNAs deposited at the miRBase 14.0 and supported by two reads of mature sequences were considered real miRNA genes.

Microarray Data Analysis: Raw data obtained in .txt files were normalized using GeneSpring GX v 12.0.  Intra array normalization was done by Quantile normalization for each chip/samples.  Inter array normalization was done by taking median of all the samples. Volcano plot based method was used to find out genes that are 2 fold differentially expressed between any 2 conditions by applying Unpaired Student T-Test for p-value calculation (p <0.05) and Benjamini-Hocheberg based FDR correction. Hierarchical clustering of differentially expressed genes was done by Pearson Uncentered algorithm with Average linkage rule to identify up and down regulated gene clusters.

Gene ontology and pathway analysis: Biological analysis of differentially expressed genes was done for Gene Ontology and Pathways using GOElite tool ([www.genmapp.org/go_elite/‎](http://www.genmapp.org/go_elite/%E2%80%8E)).  Statistically significant ontologies and pathways were filtered based on p-Value <0.05 (Obtained using Fischer Exact Test) with Benjamini Hocheberg FDR correction.

Integration of microarray and small RNA sequencing data: miRNA: mRNA integrome was performed by using differentially expressed miRNA and its target genes.  Further, miRScapeplugin ([http://ferrolab.dmi.unict.it/](http://ferrolab.dmi.unict.it/)) for Cytoscape v 8.0 was used to visualize the enriched miRNA: mRNA integrome to understand the key regulatory circuits.

**Supplementary Figures**

**Additional File 2,3. Induction of *S. aureus* induced mastitis in mice mammary tissue.**Comparison of *S. aureus*(SA1 and SA2) vs. PBS inoculated mice mammary tissuehistopathological sections from2h to 48h post infection. Scale bar shows 100μM.

**Additional File 4,5. Alteration of histone acetylation in the mice mammary tissue upon *S. aureus* infection.** Representative images of immunohistochemical analysis of mice mammary tissue (20Xmagnification, inset 40X magnification).Antibodies are indicated on the top of the panel. Biological replicates for each set of treatment (PBS, SA1 and Sa2) have been arranged in columns, which are indicated below. Scale bar 20μM.

**Additional File 6. *Staphylococcus aureus* infection specifically induces histone H3K9 and H3K14 -acetylation in mice.**SA1 and SA2 inoculated mouse mammary tissues were analyzed by Western blots using antibodies specific to acetylated H3K9, H3K14, H4K8 and H4K12, methylated H3K36 and phosphorylated H3S10. The levels of histone H3 and β-actin were used as loading controls. Lane 1,4,7, PBS inoculated (biological replicates), Lane 2,5,8, SA1 inoculated (biological replicates) and Lane 3, 6, SA2 inoculated (biological replicates) mouse mammary tissue.

**Additional File 7.** Schematic representation of small RNA data analysis workflow including integration of mRNA microarray data.

**Supplementary Figure S5. *S.aureus* infection induces IL6 and IFNγ expression in mice.**

SA1 and SA2 inoculated mouse mammary tissues were analyzed by Western blots using antibodies specific to IL6 (I) and IFNγ (II) and Tubulin(III) were used as loading controls. Lane 1, PBS inoculated, Lane 2, SA1 inoculated and Lane 3, SA2 inoculated mouse mammary tissue.

**Additional File 9:** Details of differentially expressed microRNAs in the *S. aureus* infected mice mammary tissue.

**Additional File 10:** List of differentially expressed miRNAs and expression levels of their known targets in the*S. aureus* infected mice mammary tissue.

**Additional File 11: *S. aureus* infection induces expression of IL6 and IFNg.** SA1 and SA2 inoculated mouse mammary tissues were analyzed by western blots using antibodies specific to IL6 and IFNγ and the level of α-Tubulin has been used as loading control. Lane 1, PBS inoculated, Lane 2, SA1 inoculated and Lane 3, SA2 inoculated mouse mammary tissue.

**Supplementary Table S4: Details of primers and probe sequences used for quantitative RT-PCR**

| **Gene Name** | **Forward Primer (5’-3’)** | **Reverse Primer (5’-3’)** | **Probe Cat No**  **(Universal Probe Library)** | **AMP Size** |
| --- | --- | --- | --- | --- |
| IL2 | GCTGTTGATGGACCTACAGGA | ATCCTGGGGAGTTTCAGGTT | **#15, cat.no. 04685148001** | 69 |
| IL 4 | CATCGGCATTTTGAACGAG | GACGTTTGGCACATCCATCT | **#2, cat.no. 04684982001** | 67 |
| IL 6 | ATCAGGAAATTTGCCTATTGAAA | CCAGGTAGCTATGGTACTCCAGA | **#6, cat.no. 04685032001** | 60 |
| IL 12 | CTTAGCCAGTCCCGAAACCT | TTTTCTCTGGCCGTCTTCAC | **#114, cat.no. 04693485001** | 62 |
| TNFα | AAGGGGGACCAACTCAGC | CGGACTCCGCAAAGTCTAAG | **#113, cat.no. 04693477001** | 61 |
| IFNγ | CGCTACACACTGCATCTTGG | GACTGTGCCGTGGCAGTA | **#129, cat.no. 04693655001** | 73 |
| GM CSF | TGTAGAGGCCATCAAAGAAGC | ACCTCTTCATTCAACGTGACAG | **#79, cat.no. 04689020001** | 66 |
| GAPDH | AGCTTGTCATCAACGGGAAG | TTTGATGTTAGTGGGGTCTCG | **#9, cat.no. 04685075001** | 62 |
| TLR 2 | CTGCACTGGTGTCTGGAGTC | GGGCACCTACGAGCAAGAT | #2**, cat.no. 04684982001** | 101 |
| TLR 4 | GGACTCTGATCATGGCACTG | CTGATCCATGCATTGGTAGGT | #2**, cat.no. 04684982001** | 101 |
| TLR 9 | CTCGGAACAACCTGGTGACT | ACTGGAGGCGTGAGAGATTG | #106**, cat.no. 04692250001** | 60 |
| TLR 11 | ATGGGGCTTTATCCCTTTTG | AGATGTTATTGCCACTCAACCA | #1**, cat.no. 04684974001** | 60 |
| TLR 12 | TTTCAAGCACTGGCCTAACC | GAAGCCTAGGCATGGCAGT | #31**, cat.no. 04687647001** | 60 |
| CD 14 | AAAGAAACTGAAGCCTTTCTCG | AGCAACAAGCCAAGCACAC | #26**, cat.no. 04687574001** | 89 |
| IL 1ß | TGTAATGAAAGACGGCACACC | TCTTCTTTGGGTATTGCTTGG | #78**, cat.no. 04689011001** | 68 |
| RANTES | CCTACTCCCACTCGGTCCT | GTTTCTTGGGTTTGCTGTGC | #105**, cat.no. 04692241001** | 73 |
| Lactoferrins | CGGACAGACAAGGTGGAAGT | CCATTTCTCCCAAACTGAGC | #62**, cat.no. 04688619001** | 68 |
| CXCL1 | AGACTCCAGCCACACTCCAA | TGACAGCGCAGCTCATTG | #83**, cat.no. 04689062001** | 130 |
| CXCL5 | CCTGGTCCGGGATCTTGT | CATGAATGGCGAGATGGAA | #78**, cat.no. 04689011001** | 83 |

**Supplementary Table S5. List of mRNA specific primers used for validation by qRT-PCR.**

| **Primer name** | **Primer sequence (5’-3’)** |
| --- | --- |
| CD8B-F  CD8B-R | GAAGAAGCAATGCCCGTTCC  AGGCTATCAGTGTTGTGGGC |
| CCL28-F  CCL28-R | AAGATGGGCCTCAACCTGAC  TTAGGCCTGCACTTTGGTGA |
| GSTA3-F  GSTA3-R | AAACCCAAGCAACTGCTGCC  CGTCGATCTCTACCATGGGC |
| CD14-F  CD14-R | CCTGAATTGGGCGAGAGAGG  AGCGAGTTTAGCTGACTGGG |
| RAMP1-F  RAMP1-R | TTGCAGGAAGCAGTTTCGGG  GTCGCCTGCCAATGTCAGTA |
| AQP9-F  AQP9-R | ACGCGCCTTATCCAGTTTGA  TCTACAAAGCCCACACCGTC |
| ELOVL6-F  ELOVL6-R | GGACCACTGGCCTATGACAC  CAGACCTAGTGACCGCACTG |
| FASN-F  FASN-R | TTGACGGCTCACACACCTAC  GATAAGGTCCACGGAGGCAG |
| GLRX-F  GLRX-R | TATAAAAGGGGTGGCAGGCAG  CTTCTCGGCCCCATGGTTAG |
| FCNA-F  FCNA-R | TCACAGCCAATGGGAACCAA  GCTCCATGGAACAAAGCTGC |
| CLIC6-F  CLIC6-R | AAGCTGTGGCTTCAGTCCAA  TAAGGCACACCGCTTGTGAT |
| TUBB6-F  TUBB6-R | GGTAGCTGTGTGCGACATCC  CATCATTGACCGTGGCGTC |
| AQP5-F  AQP5-R | ATTGGCTTGTCGGTCACACT  CTCAGCGAGGAGGGGAAAAG |
| APLNR-F  APLNR-R | ATGGGTGTGCATATTCCGGG  CTGGCGGAGAAAGTAGCTGT |
| S100A9-F  S100A9-R | GATGGAGCGCAGCATAACCA  GCCATCAGCATCATACACTCC |
| CCL21A-F  CCL21A-R | CATCCCGGCAATCCTGTTCT  CCTCTTGAGGGCTGTGTCTG |
| FAIM3-F  FAIM3-R | CACTCAGCAAGCAATCAGGC  AGGAGGCTCTCCTCCTTTGA |
| CCL5-F  CCL5-R | TGCTCCAATCTTGCAGTCGT  ATGCCCATTTTCCCAGGACC |
| CAMP-F  CAMP-R | CCCAAGTCTGTGAGGTTCCG  ATCTTCTCCCCACCTTTGCG |
| ITK-F  ITK-R | GGCATCAGTCCCTGAGTCAC  TGCTAAGAAGTGCAAGAATGCTG |
| POU2AF-1-F  POU2AF-1-R | AGGCGCCCCTCACTTATTTC  TGAGCTCGTACGTGTTGCTT |
| CD79B-F  CD79B-R | GCGCCAACCATAATGTCACC  AGGTGGCTGTCTGGTCAATG |
| CD3G-F  CD73G-R | CGCCAGTCAAGAGCTTCAGA  GCTTGCAGTCTACCTGTAGGG |

**Supplementary Table S6. List of miRNA specific primers used for validation by qRT-PCR.**

| **Name** | **Primer Sequence (5’-3’)** |
| --- | --- |
| mmu-miR-615-5p | CCCCGGTGCTCGGATCAT |
| mmu-miR-138-2-3p | GCTATTTCACGACACCAGGGT |
| mmu-miR-138-5p | TGGTGTTGTGAATCAGGCCG |
| mmu-miR-190-5p | CGTGTGATATGTTTGATATATTAGGT |
| mmu-miR-802-3p | CGGAGAGTCTTTGTCACTCAG |
| mmu-miR-3076-3p | ACTCTGGTCTTCCCTTGCAG |
| mmu-miR-540-5p | GGTCACCCTCTGACTCTGT |
| mmu-miR-382-5p | AAGTTGTTCGTGGTGGATTCG |
| mmu-miR-206-3p | TGGAATGTAAGGAAGTGTGTGG |
| mmu-miR-129-2-3p | AAGCCCTTACCCCAAAAAGCAT |
| mmu-miR-184-3p | GACGGAGAACTGATAAGGGTA |
| mmu-miR-1948-5p | GGTCATATGAGTATTCTGCCTAAAT |
| mmu-miR-203-3p | CGTGAAATGTTTAGGACCACTAG |
| mmu-miR-208b-3p | CTGATAAGACGAACAAAAGGTTTGT |

**Supplementary Table S7. List of primers used for ChIP-qRT-PCR.**

| Gene Name | Primer name | Primer Sequence |
| --- | --- | --- |
| AQP5 | AQP5-F  AQP5-R | CCCAAGGGCTACCACTGAGA ACGGACGGGTCAGAGTGATG |
| CCL5 | CCL5-F  CCL5-R | TAGAGGCAGAGTCATACTTC  GGCTGTGGGAGATGCATGTG |
| CD79 | CD79-F  CD79-R | GAACCCAAAGCTGTGCTACC  CTGCAGCTTGTCTCCTCTCC |
| CAMP | CAMP-F  CAMP-R | GGTTCAAGTCCCAGCACCAT TAAGTGACAGTGGCCAACCC |
| S100A9 | S100A9-F  S100A9-R | CCATTGGGAAAGAGCTACCA CTCACTTCCCTTCCATCCAA |
| CLIC1 | CLIC1-F  CLIC1-R | ATGAGTGAACACACGTCCCA TCCCCATGAGTGCCTATCTC |
| CD3G | CD3G-F  CD3G-R | CCAGAAGAACAGGCAGGAAC AGGGGTGTTGATGCTCTGAC |
| FASN | FASN-F  FASN-R | AATTGGTTTCGATGTGGAGC  CTCATCTCTGTCCTCCCACC |

**Figure S5. *S. aureus* infection induces expression of IL6 and IFNγ.** SA1 and SA2 inoculated mouse mammary tissues were analyzed by western blots using antibodies specific to IL6 and IFNγ and the level of α-Tubulin has been used as loading control. Lane 1, PBS inoculated, Lane 2, SA1 inoculated and Lane 3, SA2 inoculated mouse mammary tissue.


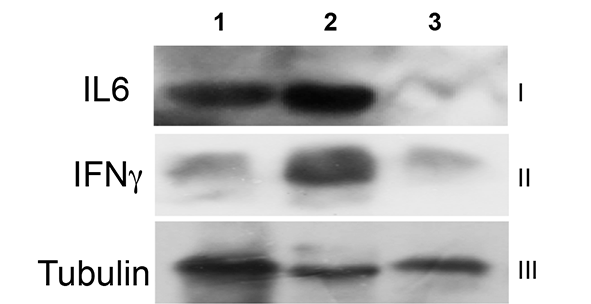

Supplement: Additional file 9 — Supplementary Results & Discussion and Methods. [file 1868-7083-6-12-S9.docx]
